# Neoadjuvant Chemotherapy Plays an Adverse Role in the Prognosis of Grade 2 Breast Cancer

**DOI:** 10.7150/jca.33168

**Published:** 2019-09-07

**Authors:** Xudong Zhu, Jinqi Xue, Xi Gu, Guanglei Chen, Fangning Cao, Huilian Shan, Dan Wang, Xinbo Qiao, Caigang Liu, Yixiao Zhang

**Affiliations:** 1 Department of Breast Surgery, Shengjing Hospital of China Medical University, Shenyang, Liaoning Province, 110004, China; 2 Yan'an Hospital of China Medical University, Yanan, Shanxi Province, 716000, China; 3 Shengjing Hospital of China Medical University, Shenyang, Liaoning Province, 110004, China

**Keywords:** Breast Cancer, NAC, Tumor Grade, pCR, Prognosis

## Abstract

**Background**: The role of neoadjuvant chemotherapy (NAC) in the prognosis of breast cancer among patients with grade 2 tumors remains unclear. As such, we aimed to explore the relationships between NAC and survival outcomes among patients with grade 2 breast cancer.

**Materials and Methods**: We collected data on 726 breast cancer patients with grade 2 tumors and at least 5-years of follow-up from the date of diagnosis. We then conducted survival analyses to examine the association between NAC and disease-free survival (DFS) and overall survival (OS). The role of NAC in prognosis was further examined in subgroup analyses, with patients stratified according to molecular subtypes, histological grade, ER status, PR status, HER2 status and Ki67 index. We also determined the main sites of local recurrence, as well as these organs involved in distant metastasis among patients receiving NAC. Finally, we analyzed independent predictive factors for DFS and OS using Cox regression analyses.

**Results**: Among patients who received NAC, the prevalence of pathologic complete response (pCR) was 9.87% (23/233), with 32.6% of patients (76/233) experiencing partial response. Survival analyses demonstrated that NAC had an overall adverse effect on DFS and OS. Subgroup analyses showed that patients who received NAC had shorter DFS in all molecular subgroups of breast cancer, with exception of triple negative breast cancer (TNBC) patients. NAC was also associated with shorter OS among patients with histological grade of 2 and a low Ki67 index. The main recurrence site was the chest well, while distant metastasis occurred in the bone, liver and lung. In Cox regression analyses, we found that NAC was an independent predictor for DFS, but not for OS.

**Conclusions**: NAC may have an adverse effect on breast cancer prognosis among patients with grade 2 tumors. These patients need not receive NAC, except when the patient has a strong desire for breast conservation, and this is unlikely to be achieved in the absence of NAC.

## Introduction

Cancer incidence is on the rise worldwide 1, and cancer is among the leading causes of death in modern society 2. Breast cancer has become increasingly common 3. A previous study of breast cancer has reported that it is the most commonly diagnosed cancer and the leading cause of cancer-related death among women 4. Advancements in the development of molecular subtypes have brought about opportunities for precision treatment of breast cancer including targeted therapies, endocrine therapies and even immune therapies 5,6. In addition, neoadjuvant chemotherapy (NAC) has played a very important role in the overall treatment strategy, particularly among women with early breast cancer 7.

The concept of NAC has evolved over the past two decades. NAC, as a term used to indicate chemotherapy administered prior to surgery, was first introduced in the 1970s. The main aim was to decrease the tumor size enough to make previously impossible surgical operations possible 8,9. Today, NAC has become a standard treatment strategy for early breast cancer, independent of T grade or tumor size 10,11. At present, the aim of NAC is not only to reduce the size of the tumor, both in the breast and the surrounding axillary tissue, in order to facilitate the operation and improve breast conservation, but also to test sensitivity to chemotherapeutic agents *in vivo*, to guide the drug selection process and improve treatment outcomes 12. Furthermore, NAC may clear micrometastases more effectively than traditional post-operation adjuvant chemotherapy 13. However, one potential pitfall of NAC is that it causes a delay in performing the surgical resection, which may increase the risk of distant metastases, thereby shortening disease-free survival (DFS), particularly among chemotherapy-resistant breast cancer patients.

A meta-analysis conducted in 2005 showed that although there was no significant difference between NAC and adjuvant chemotherapy in terms of death and DFS, patients who received NAC experienced a significantly increased rate of local recurrence compared with patients who received adjuvant chemotherapy only 14. A recent, updated meta-analysis also showed a similar result 15. The authors reported that NAC was associated with more frequent recurrence relative to adjuvant chemotherapy, with 21.4% local recurrence among patients receiving NAC, compared to 15.9% among patients receiving adjuvant chemotherapy. However, there was no significant difference in DFS or death in patients who received NAC compared to those treated with adjuvant therapy only. Overall, these findings suggest that NAC has the potential to contribute to clinical breast cancer relapse. In addition, a basic research study by Karagiannis et al found that NAC can promote breast cancer metastasis through a tumor microenvironment of metastasis (TMEM)-mediated mechanism 16. The investigators in that study found that NAC can increase the activity and density of TMEM sites in order to promote distant breast cancer metastasis. Treatment of patients with residual breast cancer using chemotherapy drugs also increased the TMEM score, as well as MENA^INV^ isoform expression, suggesting that although NAC decreased the size of the tumor, it increased the risk of metastatic dissemination. A recent study has also found that the taxanes and anthracyclines used in NAC can promote lung metastases by raising the level of tumor-derived exosomes, which were enriched for annexin A6. Annexin A6, in turn, can facilitate the establishment of breast cancer metastases in lung tissue 17. In total, these researches showed that although NAC can lower tumor grade, increase the proportion of patients eligible for breast-conserving surgery and even facilitate chemotherapy sensitivity tests in vivo, it may also have potential negative impacts on prognosis, particularly with respect to distant metastases, but possibly including death. As such, the overall role of NAC, and its impact on breast cancer prognosis, warrants further investigation. This is particularly true in China, where the proportion of patients achieving pathological complete response (pCR) or qualifying for breast-conserving surgery is well below the levels reached in Europe or North America 18. Furthermore, there are no previous papers describing the effect of NAC on prognosis among Chinese breast cancer patients. Therefore, further study is needed in order to elucidate the role of NAC in breast cancer prognosis in China.

In real-world clinical practice, breast cancer patients with grade 3 tumors have a high probability of receiving NAC in an attempt to lower the grade, grade 1 patients will likely not receive NAC. This situation was reflected in our department, in which grade 3 patients received NAC, while patients with grade 1 tumors did not. However, in patients with grade 2 tumors, some patients received NAC and some did not. At the same time, there were no clear reports on the role of NAC in breast cancer prognosis, specifically among woman with grade 2 breast cancer. Here, we performed a retrospective analysis to explore the effect of NAC on prognosis in a population of 726 Chinese breast cancer patients with grade 2 tumors. We examined whether NAC may influence patient' prognosis, whether this effect was modified by any patient characteristics, and whether there were particular metastatic sites that were most likely to be effected by NAC.

## Materials and Methods

### Patients

This study included 726 breast cancer patients treated at China Medical University between 2007 and 2014, including 233 patients who received NAC. The inclusion criteria were: 1) surgery was performed; 2) at least 10 axillary lymph nodes were dissected and evaluated; 3) the information on immunohistochemistry was complete, including ER, PR, HER2, and Ki67; 4) the administration of NAC (if applicable) and adjuvant therapy was done in accordance with Chinese clinical guidelines; 5) the surgical method was “modified radical resection of breast cancer”; and 6) the patients' tumor was grade 2. Excluded criteria were: 1) incomplete data; 2) Chemotherapeutic strategy did not meet Chinese clinical guidelines; and 3) the tumor was any grade other than 2. In addition, because nearly all patients that we could collect who received NAC underwent modified radical resection, only a few patients received breast cancer conserving surgery. Therefore, these few patients were not included in the analysis. The project was approved by the Ethics Committee of China Medical University. The institutional review board (IRB) number was 2018PS336K.

### Treatment

Modified radical resection of breast cancer was performed according to the guidelines of China. The operation included excision of the original tumor and level 1 and level 2 axillary lymph node dissection (ALND) with histological analysis. Among patients diagnosed with stage 2 breast cancer, some received anthracycline and taxane-based NAC before surgery. The NAC regimen consists of: (1) docetaxel and doxorubin every 3 weeks for 2-6 cycles; (2) doxorubin and cyclophosphamide every 3 weeks for 4 cycles; (3) epirubicin and cyclophosphamide for 4 cycles. Of note, all HER2+ breast cancer patients, in this study were long-term (5 years or more) breast cancer survivors, and most of them came from Liaoning province and could not afford the high price of trastuzumab. As a result, few of them received trastuzumab during the period of NAC. If patients included in the study had axillary lymph node metastases, radiotherapy was performed. For patients who received NAC in this study, all chemotherapy was performed prior to the surgical operation.

### Pathologic evaluation

Chinese guidelines were followed for the surgical extraction of tumors, including evaluation of histologic grade, ER status, PR status, HER2 status and the Ki67 index. Tumor grades were assigned according to the following guidelines, grade 1: tumor size≤2 cm; grade 2: 2cm < tumor size≤5 cm; grade 3: tumor size>5cm; and grade 4: tumor invaded the chest wall 19. The cut-off values for ER and PR positivity were 10% 20. Positive HER2 status was defined as a score of 3+ by IHC, or evidence of amplification by FISH 21. The cutoff value for Ki67 is 20% 22. pCR was defined as no histological evidence of malignant tumor in the primary foci of breast cancer, and no metastasis to lymph nodes. Partial response was defined as≥50% reduction in tumor size after NAC 23.

### Follow-up

Follow-up was done by breast ultrasound/MRI or other physical examination, performed every 3-6 months for the first 3 years after surgery, every 6 months in postoperative years 3-5, and every 12 months after 5 years. Additional outcomes were obtained via electronic medical records, telephone, or in-person patient/family member interview conducted in an outpatient setting. DFS was defined as the interval from the time of the operation to the time of local recurrence or distant metastasis. OS was defined as the time interval from the date of the operation to the date of death. For patients who did not experience any events, survival was calculated until the last recorded follow-up time.

### Statistical analysis

Statistical analyses were performed using SPSS 19.0. We analyzed the relationships between NAC and clinicopathological characteristics, including age, menopausal status, histological grade, ER status, PR status, HER2 status, Ki67 status, molecular subtype, local recurrence, distant metastasis, or death. Categorical data were analyzed using chi-square test or Fisher's extract test. Tumor size, positive axillary lymph node (PALN) number, DFS, and OS were analyzed using an independent sample t test. The Kaplan-Meier method was used to estimate survival curves, and the log-rank test was used to compare survival outcomes. Univariate and multivariate Cox regression analyses using the enter method were conducted to assess potential predictors of DFS and OS, including estimated hazard ratios (HRs) and 95% confidence intervals (CIs). All *p* values are two-sided, and *p<0.05* was defined as statistically significant.

## Results

### Basic clinicopathological characteristics

A total of 726 breast cancer patients with grade 2 tumors were included in this study, including 233 patients who were treated with NAC. Among patients who received NAC, 9.87% (23/233) achieved pCR, and 32.6% (76/233) experienced a partial respond. These proportions were lower than previously published results 16. **Table 1** shows the relationships between treatment with NAC and various clinicopathological characteristics. Patients ranged in age from 22 to 81 years, with an average of 50.42 years. In the initial analysis, we found that NAC was not associated with any basic clinicopathological characteristics (All *p* values>0.05). Among the 493 patients who did not receive NAC, 11 patients experienced a local recurrence (2.2%). 54 patients had distant metastases (11.0%), and 32 patients died (6.5%) during the study period. Among patients who did receive NAC, 12 patients experienced a local recurrence (4.8%). 51 patients had distant metastases (21.9%), and 18 patients died (7.7%). There were significant differences between the NAC group and the no NAC group with respect to both local recurrence and distant metastasis (*p*= 0.036 and *p*<0.001, respectively). However, there was no significant difference in mortality (*p*= 0.540). The average DFS and OS were 57.41 months and 70.13 months, respectively in patients who received NAC, and 75.46 months and 82.13 months, respectively in patients not treated with NAC, and these differences were statistically significant (both *p*<0.001). From these results, we can conclude that, among patients with grade 2 tumors, treatment with NAC may lead to poorer prognosis.

### Survival analysis by NAC treatment

**Figure 1:**
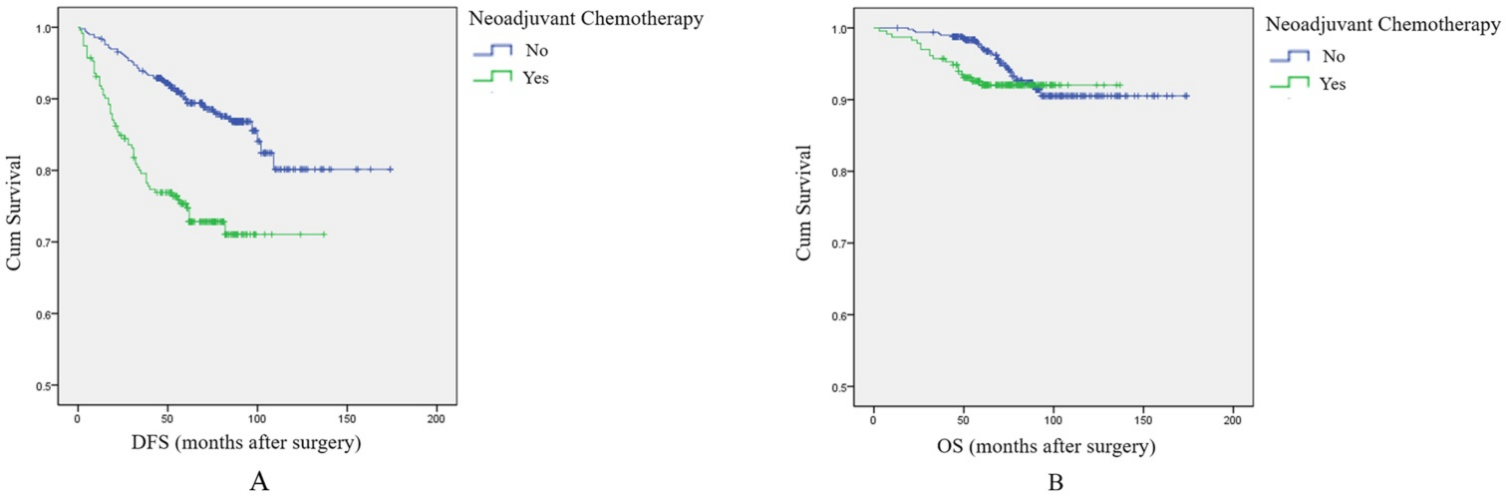
** The effect of NAC on prognosis among breast cancer patients overall.** A: Survival curve for DFS in breast cancer patients according to NAC treatment. B: Survival curve for OS in breast cancer patients according to NAC treatment.

Based on the above results, we included all patients in an analysis of the effect of NAC on DFS and OS. Survival analysis showed that patients receiving NAC had shorter DFS and OS compared with patients not treated with NAC. The difference was significant for DFS, but not for OS (*p*<0.001, log-rank test, Figure 1A; and *p*=0.236, log-rank test, Figure 1B, respectively). The results also showed that among patients with grade 2 tumors, although NAC can shrink tumors and facilitate surgical resection, it may also contribute to local recurrence, distant metastasis, and even death.

### NAC had a negative effect on the prognosis of different molecular subtype breast cancer patients

To further explore the effect of NAC on breast cancer prognosis, we stratified the population according to molecular subtype and conducted additional survival analyses.

**Figure 2:**
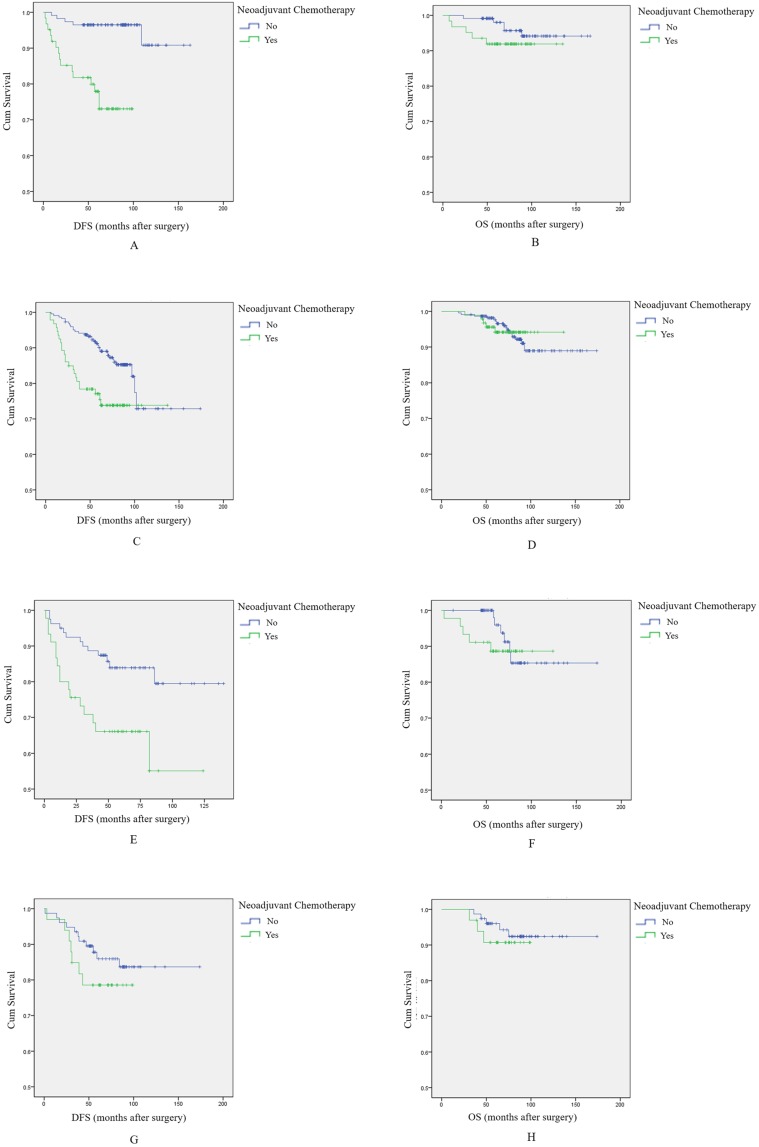
** The effect of NAC on prognosis among breast cancer patients with different molecular subtypes.** A: Survival curve for DFS in luminal A type breast cancer patients according to NAC treatment. B: Survival curve for OS in luminal A type breast cancer patients according to NAC treatment. C: Survival curve for DFS in luminal B type breast cancer patients according to NAC treatment. D: Survival curve for OS in luminal B type breast cancer patients according to NAC treatment. E: Survival curve for DFS in HER2+ breast cancer patients according to NAC treatment. F: Survival curve for OS in HER2+ breast cancer patients according to NAC treatment. G: Survival curve for DFS in TNBC patients according to NAC treatment. H: Survival curve for OS in TNBC patients according to NAC treatment.

Among 176 Luminal A type breast cancer patients, survival analysis showed that NAC was associated with shorter DFS and OS, and again the difference was significant for DFS, but not for OS (log-rank test, *p*<0.001, Figure 2A; and *p*=0.236, Figure 2B, respectively).

Among 315 patients with Luminal B type breast tumors, NAC was associated with shorter DFS (*p*=0.004, log-rank test, Figure 2C). There was no difference in OS between patients treated with NAC and those who were not (*p*=0.999, log-rank test, Figure 2D).

Among 125 HER2+ breast cancer patients, patients receiving NAC again had significantly shorter DFS (*p*=0.006, log-rank test, Figure 2E), while there was no relationship with OS (*p*=0.503, log-rank test, Figure 2F).

Finally, among 110 triple negative breast cancer (TNBC) patients, patients who receive NAC had nominally shorter DFS and OS, but differences were not statistically significant (*p*=0.305, log-rank test, Figure 2G; *p*=0.551, log-rank test, Figure 2H).

In total, these results indicate that NAC had a negative effect on DFS, regardless of breast cancer subtype. However, there was no clear association with OS.

### Additional subgroup analyses

To further explore the role of NAC on breast cancer patients' prognosis, we divided patients into five separate subgroups based on histological grade, ER status, PR status, HER2 status and Ki67 index.

**Figure 3:**
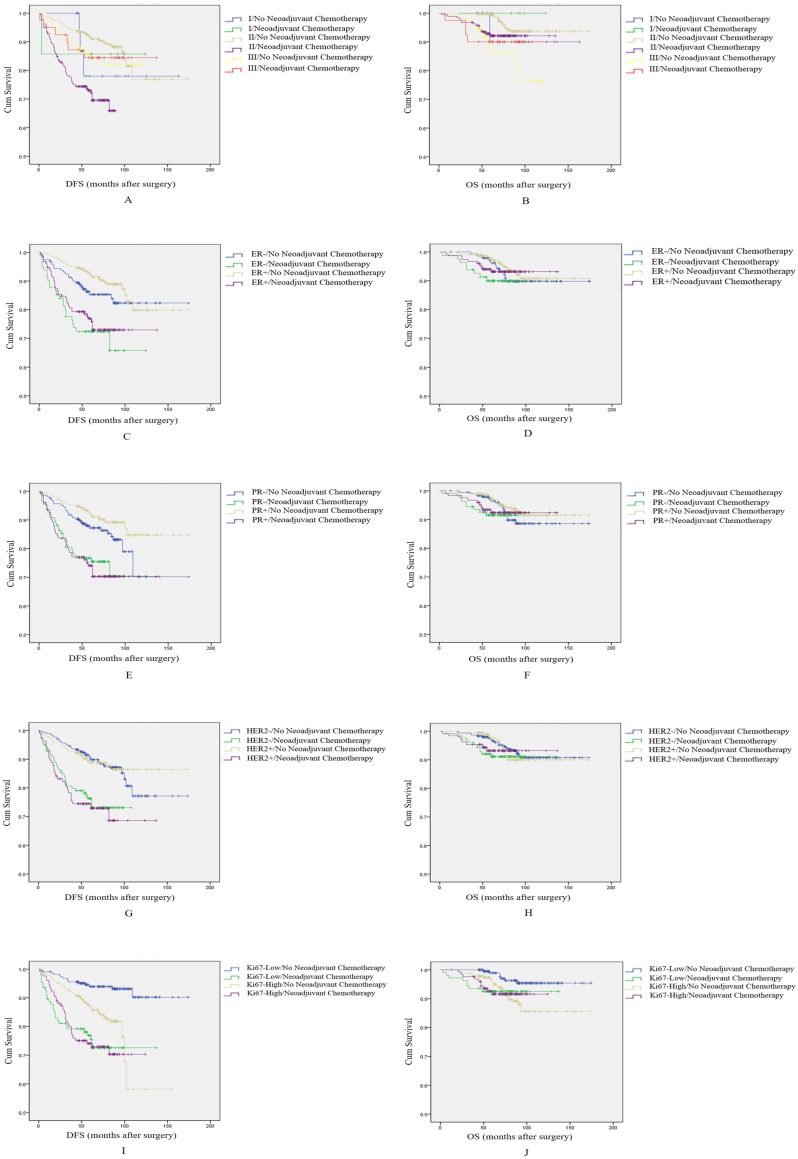
** Subgroup analysis to explore the effect of NAC on breast cancer patients' prognosis.** A: Survival curve for DFS by histological grade according to NAC treatment. B: Survival curve for OS by histological grade according to NAC treatment. C: Survival curve for DFS by ER status according to NAC treatment. D: Survival curve for OS by ER status according to NAC treatment. E: Survival curve for DFS by PR status according to NAC treatment. F: Survival curve for OS by PR status according to NAC treatment. G: Survival curve for DFS by HER2 status according to NAC treatment. H: Survival curve for OS by HER2 status according to NAC treatment. I: Survival curve for DFS by Ki67 status according to NAC treatment. J: Survival curve for OS by Ki67 status according to NAC treatment.

In survival analyses by histological grade, we find that NAC had a negative impact on DFS and OS in patients with histological grade 2 (*p*<0.001, log-rank test, Figure 3A; and *P*=0.028, log-rank test, Figure 3B, respectively). However, among patients with a histological grade of 1 or 3, NAC did not influence DFS or OS (*p*=0.802 and *p*=0.403, respectively for patients with histological grade of 1; and *p*=0.882 and *p*=0.376, respectively in patients with a histological grade of 3).

In the subgroup based on ER, patients who received NAC had a shorter DFS, and this difference was significant for both ER+ and ER- patients (*p*=0.004, log-rank test, Figure 3C; and *p*<0.001, log-rank test, Figure 3C, respectively). However, there was no significant difference in OS among patients with ER- or ER+ tumors (*p*=0.349, log-rank test, Figure 3D; and *P*=0.495, log-rank test, Figure 3D, respectively). Of note, we also found that among patients who received NAC, ER+ patients had a longer DFS and OS than patients with ER-. As such, ER positive status may attenuate the negative effect of NAC on prognosis.

In the subgroup based on PR, the effect of NAC on patient' prognosis was the same as the ER group; patients who received NAC had a shorter DFS among patients with PR- or PR+ status (*p*=0.008, log-rank test, Figure 3E; and *p*<0.001, log-rank test, Figure 3E, respectively). NAC again not have an effect on OS among these two groups of patients (*p*=0.453, log-rank test, Figure 3F; and *p*=0.368, log-rank test, Figure 3F, respectively). However, in this case, the effect of NAC on prognosis was not modified by PR status.

In the subgroup based on HER2, patients who received NAC had slightly shorter DFS. The difference was significant among these two groups (*p*<0.001, log-rank test, Figure 3G; and *P*<0.001, log-rank test, Figure 3G, respectively). The effect on OS was again not significant among these two groups of patients (*p*=0.240, log-rank test, Figure 3H; and *p*=0.695, log-rank test, Figure 3H, respectively). HER2 status also did not modify the effect of NAC on prognosis.

Finally, in the subgroup based on Ki67 index, patients who received NAC had shorter DFS than patients without NAC, among both patients with low Ki67 index and high Ki67 index. The difference was significant (*p*<0.001, log-rank test, Figure 3I; and *p*=0.006, log-rank test, Figure 3I, respectively). Among patients with a low Ki67 index, NAC had a negative effect on OS (*p*=0.036, log-rank test, Figure 3J). However, in patients with a high Ki67 index, NAC had no effect (*p*=0.989, log-rank test, Figure 3J). At the same time, from analyzing the survival curves it is apparent that patients who received NAC and had a high Ki67 index may have shorter DFS relative to patients with low Ki67 index. As such, high Ki67 index may contribute to the adverse effect of NAC on DFS.

### Organs and sites of local recurrence and distant metastasis in breast cancer patients who received NAC

In Luminal A type breast cancer patients, the main sites of local recurrence were axillary lymph nodes (1/15), chest wall (1/15), neck (1/15), and upper clavicle lymph nodes (1/15). The top 3 distant metastasis organs were bone (8/15), liver (4/15), and lung (2/15). In Luminal B type breast cancer patients, the main sites of local recurrence were chest wall (4/23), and upper clavicle lymph nodes (3/23).

The top 3 distant metastasis organs were bone (11/23), liver (6/23), and lung (5/23). In the HER2+ breast cancer patients, the main site of local recurrence was chest wall (5/16). The top 3 distant metastasis organs were lung (6/16), liver (5/16) and bone (1/16). In TNBC patients, the main site of local recurrence also was chest wall (3/7). The top 3 distant metastasis organs were bone (3/7), lung (2/7), and brain (1/7). The specific sites of local recurrence and organs of distant metastasis for all patients are shown in **Table 2**. Overall, the main local recurrence site in patients who received NAC was the chest wall. The main organs of distant metastasis were bone, liver, and lung. Among patients who receive NAC, these positions of recurrence or organs involved in metastasis should be monitored carefully in order to improve prognosis.

### Predictive factors associated with prognosis

Univariate and multivariate cox regression analyses were used to evaluate if NAC were associated with DFS and OS among these 726 breast cancer patients (**Table 3** and **Table 4**).

For DFS, the univariate cox regression analysis showed that tumor size, PALN number, Ki67 status, molecular subtype, and NAC were associated with DFS (these *p* values were shown in **Table 3**). These factors were entered a multivariate cox regression analysis, which found that among these patients, NAC were independent predictors of DFS in these patients (*p*<0.001).

As for OS, univariate cox regression analysis showed that tumor size, PALN number, histological grade 1, and Ki67 index were associated with OS (*p*<0.001, *p*<0.001, *p=*0.007, and *p=*0.028, respectively). These variables were further analyzed in a multivariate cox regression analysis, which found that tumor size and PALN number were independent predictive factors for OS (*p*=0.021 and *p=*0.001), but NAC was not. These results were shown in **Table 4**.

## Discussion

In this study, we found that NAC may have an adverse effect on prognosis among breast cancer patients with grade 2 tumors, particularly with respect to DFS. We compared the relationships between NAC treatment and local recurrence, distant metastasis, and death, and found that NAC was associated with overall poorer prognosis, including shorter DFS and OS. Survival analysis showed that NAC had an adverse effect on DFS in patients with grade 2 tumors. We further divided these patients into subgroups according to: molecular subtypes, histological grade, ER-/+, PR-/+, HER2-/+, and Ki67 high or low index, in order to analyze the effect of NAC on prognosis. Patients who received NAC had a shorter DFS in all subgroups except among TNBC patients, and shorter OS in patients with histological grade of 2 and a low Ki67 index. Among patients who received NAC, patients with ER- and high Ki67 index tumors may have a worse prognosis. As such, these patients may experience worse survival outcomes in response to NAC treatment. The main site of recurrence in these patients was the chest wall. The main metastasis organs were bone, liver, and lung. We also found that NAC were independent predictors for DFS, but not for OS.

The present clinical study showed that NAC can increase the proportion of patients who experience local recurrence, but may not affect distant metastasis or death 24,25,26. One possible explanation for this difference may be the proportion of patients who receive pCR, as higher rates of pCR are associated with better prognosis 27. The prevalence of pCR reported in previous breast cancer patients varied from 28% to 40%. Including patients with a partial response, the overall prevalence of response may vary from 60%-80% 28,29. However, in Chinese breast cancer patients, the prevalence of complete and partial pCR was significantly lower than that in other patients outside of China. Like the results of our study, the prevalence of pCR in Chinese breast cancer may be 10%-15% 30. The lower pCR may be one of the reasons for the higher ratio of local recurrence and distant metastasis. If surgery was delayed and pCR was still not achieved, these patients may have poor prognoses. The second reason may disparate effect of NAC on patients with grade 2 tumors. In these patients, NAC can contribute to local recurrence and distant metastasis. Further, the difference in OS by NAC administration was not significant, suggesting that NAC cannot extend survival time. Since surgery can be performed in these patients, even without shrinking the tumor size, the application of NAC may unnecessarily delay the operation, contributing to distant metastasis and leading to a worse prognosis.

The present studies contributed to our understanding of the prevalence of local recurrence and the performance of breast-conserving surgery 15. In Chinese breast cancer patients, the prevalence of breast-conserving surgery is very low, and the main aim of NAC is to improve surgical outcomes 31. As such, in the patients in our study, the main reason for the high rate of local recurrence, distant metastasis, and death may be due to the use of NAC in patients with grade 2 tumors, and the lower rate of pCR.

This paper also has some limitations. First, this was a retrospective analysis, which has inherent shortcomings relative to a clinical trial. Second, we could only collect patients undergoing modified radical resection. We did not include patients undergoing breast-conserving surgery owing to the small number of these patients. In the future, a larger randomized controlled trial needs to be performed to validate these conclusions, including patients with breast-conserving surgery.

Although NAC can increase the rate of local recurrence, distant metastasis, and death to some degree, patients with grade 3 or more tumors or patients who have a strong desire to qualify for breast-conserving surgery, may still benefit from NAC. As such, doctors may appropriately extend the time of NAC to try to reach pCR, an outcome which is known to improve prognosis 27.

In conclusion, our results showed that NAC may have an adverse effect on prognosis among breast cancer patients with grade 2 tumors. Possible explanations for this association were that NAC delayed surgical operation, these patients often did not achieve a pCR, and NAC may have contributed to local recurrence and distant metastasis, all without improving survival time. As such, patients with grade 2 tumors need not receive NAC, except in the specific circumstance in which breast conservation is highly valued, and the patient is unlikely to be a candidate for breast-conserving surgery in the absence of NAC.

## Figures and Tables

**Table 1:** Correlations between neoadjuvant chemotherapy and clinicopathological characteristics

Variables	Neoadjuvant Chemotherapy (%)	No Neoadjuvant Chemotherapy (%)	*P*-value
No. of Patients	233(32.1)	493(67.9)	
Age (year)			0.549
≤45	52(22.3)	120(24.3)	
>45	181(77.7)	373(75.7)	
Menopausal status			0.580
Premenopausal	128(54.9)	260(52.7)	
Postmenopausal	105(45.1)	233(47.3)	
Tumor size (cm)			0.421
Median (range)	2.70(2.0-5.0)	2.67(2.0-5.0)	
PALN Number			0.833
Median (range)	1.96(0-17)	1.92(0-16)	
Histological grade			0.757
I	7(3.0)	19(38.5)	
II	186(82.8)	383(77.7)	
III	40(17.2)	91(18.5)	
ER Status			0.574
Positive	152(65.3)	332(67.3)	
Negative	81(34.8)	161(32.7)	
PR Status			0.224
Positive	124(53.2)	286(58.0)	
Negative	109(46.8)	207(42.0)	
HER2 Status			0.084
Positive	107(45.9)	193(39.1)	
Negative	126(54.1)	300(60.9)	
Ki67 Index			0.943
>20	126(54.1)	268(54.4)	
≤20	107(45.9)	225(45.6)	
Molecular Subtype			0.416
Luminal A	62(26.6)	114(23.1)	
Luminal B	93(39.9)	222(45.1)	
HER2+	45(19.3)	80(16.2)	
TNBC	33(14.2)	77(15.6)	
Local Recurrence			0.036
Yes	12(5.2)	11(2.2)	
No	221(94.8)	482(97.8)	
Distant Metastasis			<0.001
Yes	51(21.9)	54(11.0)	
No	182(78.1)	439(89.0)	
DFS (month)			<0.001
Median (range)	57.41(1-137)	75.46(1-174)	
Death			0.540
Yes	18(7.7)	32(6.5)	
No	215(92.3)	461(93.5)	
OS (month)			<0.001
Median (range)	70.13(3-137)	82.13(13-174)	

**Table 2:** The organs of distant metastasis or sites of local recurrence happened in different molecular subtype breast cancer patients received neoadjuvant chemotherapy

Organs/Sites	Luminal A (%)	Luminal B (%)	HER2+ (%)	TNBC (%)
No. of Patients	15(24.6)	23(37.7)	16(26.2)	7(11.5)
Lung	2(13.3)	5(21.7)	6(37.5)	2(28.6)
Liver	4(26.7)	6(26.1)	5(31.3)	0
Bone	8(53.3)	11(47.8)	1(5.3)	3(42.9)
Brain	1(6.7)	1(4.3)	1(5.3)	1(14.3)
Axillary lymph nodes	1(6.7)	1(4.3)	1(5.3)	0
Chest wall	1(6.7)	4(17.4)	5(31.3)	3(42.9)
Neck	1(6.7)	0	0	0
Upper clavicle lymph nodes	1(6.7)	3(13.0)	1(6.3)	2(28.6)
Lower clavicle lymph nodes	0	1(4.3)	0	0
Cervical lymph nodes	0	1(4.3)	0	0
Mediastinal diaphragm	0	1(4.3)	1(6.3)	2(28.6)
Colon	0	1(4.3)	0	0
Axillary	0	1(4.3)	1(6.3)	0
Mediastinal lymph nodes	0	0	1(6.3)	0
Adrenal gland	0	0	0	1(14.3)

**Table 3:** Univariate and multivariate cox regression analyses of clinicopathological risk factors for disease-free survival among these patients

Variables		DFS		
	Univariate analysis		Multivariate analysis	
	HR (95%CI)	*P*-value	HR (95%CI)	*P*-value
Age	0.807(0.543-1.199)	0.288	NA	
Menopausal status	1.114(0.931-1.334)	0.239	NA	
Tumor size(cm)	2.743(2.253-3.338)	<0.001	2.626(2.040-3.380)	<0.001
PALN Number	1.209(1.158-1.263)	<0.001	1.138(1.083-1.196)	<0.001
Histological grade			NA	
I		0.866		
II	1.145(0.421-3.116)	0.791		
III	1.016(0.347-2.972)	0.977		
ER Status	0.718(0.498-1.034)	0.075	NA	
PR Status	1.134(0.949-1.355)	0.167	NA	
HER2 Status	0.950(0.793-1.137)	0.573	NA	
Ki67 Index	0.768(0.636-0.928)	0.006	NS	
Molecular Subtype				
Luminal A		0.017	NS	
Luminal B	0.641(0.444-0.925)	0.017	NS	
HER2+	1.007(0.769-1.320)	0.959	NS	
TNBC	1.599(1.157-2.210)	0.004	NS	
Neoadjuvant chemotherapy	2.644(1.845-3.787)	<0.001	3.458(2.339-5.110)	<0.001

NA: Non-analysis, NS: Non-significant

**Table 4:** Univariate and multivariate cox regression analyses of clinicopathological risk factors for overall survival among these patients

Variables		OS		
	Univariate analysis		Multivariate analysis	
	HR (95%CI)	*P*-value	HR (95%CI)	*P*-value
Age	1.109(0.568-2.167)	0.761	NA	
Menopausal status	1.137(0.857-1.509)	0.374	NA	
Tumor size(cm)	2.047(1.510-2.774)	<0.001	1.530(1.066-2.197)	0.021
PALN Number	1.206(1.135-1.281)	<0.001	1.126(1.050-1.207)	0.001
Histological grade			NS	
I		0.007		
II	1.391(0.190-10.190)	0.745		
III	3.533(0.470-26.555)	0.220		
ER Status	0.714(0.403-1.265)	0.248	NA	
PR Status	1.180(0.894-1.559)	0.243	NA	
HER2 Status	0.982(0.738-1.308)	0.903	NA	
Ki67 Index	0.712(0.526-0.964)	0.028	NS	
Molecular Subtype			NA	
Luminal A		0.449		
Luminal B	0.754(0.444-1.280)	0.296		
HER2+	0.897(0.587-1.369)	0.614		
TNBC	1.471(0.880-2.458)	0.141		
Neoadjuvant chemotherapy	1.419(0.793-2.538)	0.238	NA	

NA: Non-analysis, NS: Non-significant

## Abbreviations

NAC: neoadjuvant chemotherapy
DFS: disease-free survival
OS: overall survival
TNBC: triple negative breast cancer
TMEM: tumor microenvironment of metastasis
pCR: pathological complete response
IRB: institutional review board
ALND: axillary lymph node dissection
PALN: positive axillary lymph node
HRs: hazard ratios
CIs: confidence intervals
